# Lung injury prediction score for the emergency department: first step towards prevention in patients at risk

**DOI:** 10.1186/1865-1380-5-33

**Published:** 2012-09-03

**Authors:** Marie-Carmelle Elie-Turenne, Peter C Hou, Aya Mitani, Jonathan M Barry, Erica Y Kao, Jason E Cohen, Gyorgy Frendl, Ognjen Gajic, Nina T Gentile

**Affiliations:** 1 Department of Emergency Medicine, University of Florida College of Medicine, PO Box 100186, 1329 SW 16th Street, Gainesville, FL 32610, USA; 2 Emergency Department, Shands University of Florida Medical Center, Gainesville, FL, USA; 3 Department of Emergency Medicine, Brigham and Women’s Hospital, 75 Francis Street, Boston, MA 02115, USA; 4 Division of Burn, Trauma, and Surgical Critical Care, Brigham and Women’s Hospital, Boston, MA, USA; 5 Surgical Intensive Care Unit Translational Research (STAR) Center, Brigham and Women’s Hospital, Boston, MA, USA; 6 Harvard Medical School, Department of Emergency Medicine & Division of Burn, Trauma, and Surgical Critical Care, Department of Surgery, Brigham and Women’s Hospital, 75 Francis Street, Neville House 312-B, Boston, MA 02115, USA; 7 Department of Anesthesiology, Perioperative and Pain Medicine, Brigham and Women’s Hospital, Boston, MA, USA; 8 Department of Emergency Medicine, Albany Medical Center, Albany, NY, USA; 9 Albany Medical College, Albany, NY, USA; 10 Department of Medicine, Division of Pulmonary and Critical Care Medicine, Mayo Clinic, Rochester, MN, USA; 11 Multidisciplinary Epidemiology and Translational Research in Intensive Care (METRIC), Mayo Clinic, Rochester, MN, USA; 12 Mayo Medical School, Rochester, MA, USA; 13 Department of Emergency Medicine, Temple School of Medicine, Philadelphia, PA, USA; 14 Department of Medicine, Stanford Hospitals and Clinincs, 300 Pasteur Drive, Room: S102, MC: 5110, Stanford, CA 94305, USA; 15 F. Edward Hebert School of Medicine, Uniform Services University of the Health Sciences, 4301 Jones Bridge Road, Bethesda, MD 20814-4712, USA; 16 Albany Medical Center Emergency Medicine Group, 47 New Scotland Avenue, MC 139, Albany, NY 12208, USA; 17 Department of Anesthesiology Perioperative and Pain Medicine, Brigham and Women’s Hospital, 75 Francis Street, Boston, MA 02115, USA; 18 Mayo Clinic, Pulmonary and Critical Care Medicine, Old Marian Hall, Second Floor, Room 115, 200 First St. SW, Rochester, MN 5590, USA

## Abstract

**Background:**

Early identification of patients at risk of developing acute lung injury (ALI) is critical for potential preventive strategies. We aimed to derive and validate an acute lung injury prediction score (EDLIPS) in a multicenter sample of emergency department (ED) patients.

**Methods:**

We performed a subgroup analysis of 4,361 ED patients enrolled in the previously reported multicenter observational study. ED risk factors and conditions associated with subsequent ALI development were identified and included in the EDLIPS model. Scores were derived and validated using logistic regression analyses. The model was assessed with the area under the receiver-operating curve (AUC) and compared to the original LIPS model (derived from a population of elective high-risk surgical and ED patients) and the Acute Physiology and Chronic Health Evaluation (APACHE II) score.

**Results:**

The incidence of ALI was 7.0% (303/4361). EDLIPS discriminated patients who developed ALI from those who did not with an AUC of 0.78 (95% CI 0.75, 0.82), better than the APACHE II AUC 0.70 (*p* ≤ 0.001) and similar to the original LIPS score AUC 0.80 (*p* = 0.07). At an EDLIPS cutoff of 5 (range −0.5, 15) positive and negative likelihood ratios (95% CI) for ALI development were 2.74 (2.43, 3.07) and 0.39 (0.30, 0.49), respectively, with a sensitivity 0.72(0.64, 0.78), specificity 0.74 (0.72, 0.76), and positive and negative predictive value of 0.18 (0.15, 0.21) and 0.97 (0.96, 0.98).

**Conclusion:**

EDLIPS may help identify patients at risk for ALI development early in the course of their ED presentation. This novel model may detect at-risk patients for treatment optimization and identify potential patients for ALI prevention trials.

## Background

Acute lung injury (ALI) is widely recognized as an important cause of poor outcome in critically ill patients. Acute respiratory distress syndrome (ARDS), a severe variant of ALI, was originally described in 1967 by Ashbaugh et al. as the acute onset of tachypnea, hypoxemia, and poor pulmonary compliance resistant to traditional medical therapies. At the time, the authors suggested that the use of positive end expiratory pressure and corticosteroids may be of utility [1]. This prompted investigations and dialogue of an international scale that established standardized parameters used to describe ALI, the 1994 American European Consensus Conference criteria: acute hypoxemia with a ratio of the partial pressure of arterial oxygen to the fraction of inspired oxygen (PaO_2_:FiO_2_) of 300 mmHg or less (ARDS is defined as PaO_2_:FiO_2_ 200 mmHg or less), bilateral infiltrates seen on a frontal chest radiograph that are consistent with pulmonary edema, and no clinical evidence of left atrial hypertension [2].

Traditionally, ALI has been generally recognized as an intensive care unit (ICU)condition; a complication of protracted illness during an inpatient stay, infrequently diagnosed in the emergency department (ED) [3]. Numerous reports, however, have revealed that ALI development is often early, with 50% of cases occurring within the first 24 h of admission [4]. Among ED populations, it has been documented to develop within hours of initial presentation [5-7]. Most commonly, patients are resuscitated for respiratory distress and subsequently experience a precipitous decline in clinical status prompting intubation, mechanical ventilation, the use of increasing doses of supplemental oxygen, and positive end expiratory pressure (PEEP). Because of similarities in presentation, these ALI cases may have been difficult to diagnose and were managed as cardiogenic pulmonary edema [5].

The source of the initiating insult in ALI may be pulmonary (i.e., aspiration, pneumonia) or extrapulmonary (sepsis, shock, pancreatitis) in origin. The pathophysiology involves the disruption of the alveolar capillary interface, resulting in the extravasation of protein-rich fluid into alveoli, the induction of local inflammatory mediators, and hypercoagulability [8-10]. The clinical picture is characterized by profound hypoxemia, ventilation perfusion mismatch, and restrictive lung disease [11]. The outcome is frequently prolonged mechanical ventilation and ICU length of stay, and ultimately death. Even previously healthy survivors have long-term physical and cognitive impairment [12].

As of the date of this publication, a PubMed database search of the literature yields over 25,000 articles published since 1967 with keywords acute lung injury or acute respiratory distress syndrome. Despite countless large-scale investigations, ALI and ARDS affect well over 200,000 persons in the US annually, few beneficial treatments have emerged, and the mortality rate is 38-44% [13,14].

To date, supportive care with the use of a low tidal volume ventilation strategy remains the sole effective therapeutic measure for ALI [15]. Recognizing the paucity of therapies, investigators have posited whether a role for preventive strategies may exist to curb the progression to ALI in the at-risk patient.

Previous research of ALI has largely been limited to ICU populations; hence, recruitment into study protocols often occurs well after the diagnosis of ALI has been established [3,16]. This approach likely identifies patients that are beyond the pre-morbid window of intervention. The presentation of known predispositions to ALI such as pneumonia, sepsis, shock, and trauma to emergency rooms may provide opportunities to limit a patient’s risk of developing downstream direct and indirect pulmonary insults. Hence, reliable identification and risk stratification of ED patients for ALI may prove a viable approach for early goal-directed interventions and preventive measures.

While the pathophysiology of ALI is well documented, models predicting the risk for developing ALI are not well established. While scores have been developed from mixed populations, none has been derived and validated in an exclusively emergency department population [17]. The purpose of this study is to develop a model using readily accessible clinical data for the identification of ED patients at risk for ALI. Using a previously published prediction model for ALI, the Lung Injury Prediction Score (LIPS), we perform a multi-center derivation, model refinement (EDLIPS), and validation study of emergency department patients presenting with predisposing risk factors of ALI previously identified in the literature [17].

## Methods

### Study design

This is a subgroup analysis of data from a multicenter, observational cohort study, the United States Critical Injury and Illness Trial Group - Lung Injury Prevention Study 1 (USCIITG-LIPS 1). Each participating center sought approval from its local institutional review board.

### Study setting

From March through August 2009, 22 centers (20 American and 2 Turkish hospitals) enrolled patients with at least one ALI predisposition admitted from the ED. Patients were enrolled prospectively at 19 study sites and retrospectively at 3 sites.

### Selection of participants

Consecutive adult ED patients admitted to academic and community acute care hospitals were eligible for the study if they presented with one or more study defined ALI predisposing conditions. Patients were excluded if they presented with ALI at initial assessment, transferred from an in-patient setting, died in the ED, were admitted for comfort or hospice care, or were re-admitted during the study period. Hospital admission logs were reviewed to minimize the possibility that patients with predisposing condition were missed. After identification of at-risk ED patients, they were followed through their hospitalizations prospectively in 19 hospitals. In three hospitals that enrolled retrospectively, investigators followed the same protocol and definitions, but data were collected after patient discharge.

### Data collection and processing

To derive and validate the proposed EDLIPS prediction model, a subgroup analysis of a larger prospective cohort study was performed [17]. Baseline characteristics including demographics, co-morbidities, and clinical variables were collected during the first 6 h of ED evaluation. We used clinical variables previously documented in the literature associated with the development of ALI [3,18-21]. Each patient was screened for 24 predisposing conditions and ALI risk modifiers. Predisposing conditions included shock, aspiration, sepsis, pneumonia, acute abdomen, high-risk trauma (traumatic brain injury, smoke inhalation, near drowning, lung contusion, multiple fractures), and necessity for emergency and high-risk surgeries (thoracic, spine, abdominal, cardiac, aortic vascular). ALI risk modifiers included alcohol abuse, obesity, chemotherapy, diabetes mellitus, smoking, tachypnea, hypoxemia, oxygen supplementation, hypoalbuminemia, and acidosis. During data collection, a specific definition of each clinical variable was explicitly outlined. The study outcome used to derive the prediction rule was the diagnosis of ALI.

De-identified subject information was entered at each center into the secure, password-protected NIH-supported web form (REDCap http://www.project-redcap.org). Electronic range checks and validation rules were utilized to eliminate erroneous data entry and artifacts in numeric values. Prior to study initiation at each site, investigators and study coordinators reviewed structured online training (http://depts.washington.edu/kclip/about.shtml) for ALI assessment and for definitions of each risk factor (see Appendix 1). In addition, a formal training session was provided during the 2009 USCIITG meeting in Nashville, TN. The principal investigators from each site provided a written statement stating their responsibility for the quality control of data collection and entry.

### Data analysis

All clinical variables were collected for each patient. The criterion for diagnosis of ALI was derived from the American-European Consensus Conference definition: bilateral pulmonary infiltrates and hypoxemia (ALI: PaO_2_/FIO_2_ < 300; ARDS: PaO_2_/ FIO_2_ < 200) in the absence of clinical signs of left atrial hypertension as the main explanation for pulmonary edema.

The primary analysis consisted of a validation of the predictive ability of the EDLIPS model, modified from the previously validated LIPS model derived in a diverse multi-disciplinary cohort [17]. All emergency department patients were included in the sub-cohort analyses. Any missing data were treated as an absent disease state or a normal variable. To simplify the calculation, variables with minimal or no effect size were removed (i.e., pancreatitis, alcohol abuse, smoking, and tachypnea). Variables identified to be present in fewer than ten patients were also removed (i.e., near drowning, thoracic surgery).

EDLIPS weighting points were adjusted based on logistic regression analysis results from a training data set (a random sample of 2,000 patients from the cohort). If statistically significant (*p* < 0.05), the EDLIPS point value was derived by doubling the parameter estimate and rounding to the closest 0.5. The variables with less robust *p*-values (*p* > 0.05) had parameter estimates rounded to the closest 0.5. Subsequently, the model was independently validated in the remaining patients (validation cohort of 2,361). Model discrimination was assessed by calculating the area under the receiver operating characteristic curve (AUC). The threshold score providing the best combination of sensitivity and specificity was determined by AUC analysis. Corresponding positive and negative predictive values, positive and negative likelihood ratios, and their 95% CIs were calculated. A sensitivity analysis was performed to determine the model performance at different cutoff points.

In secondary analyses, to determine the mortality burden due to the development of ALI, we performed a logistic regression analysis adjusted for ALI development, EDLIPS, and baseline severity of illness (Acute Physiology and Chronic Health Evaluation [APACHE] II score). In addition, we compared the performance of EDLIPS to the original LIPS previously described in the literature [17]. All statistical analysis was operated in SAS 9.2 (SAS Institute, Cary, NC).

## Results

### Characteristic of study subjects

**Figure 1:**
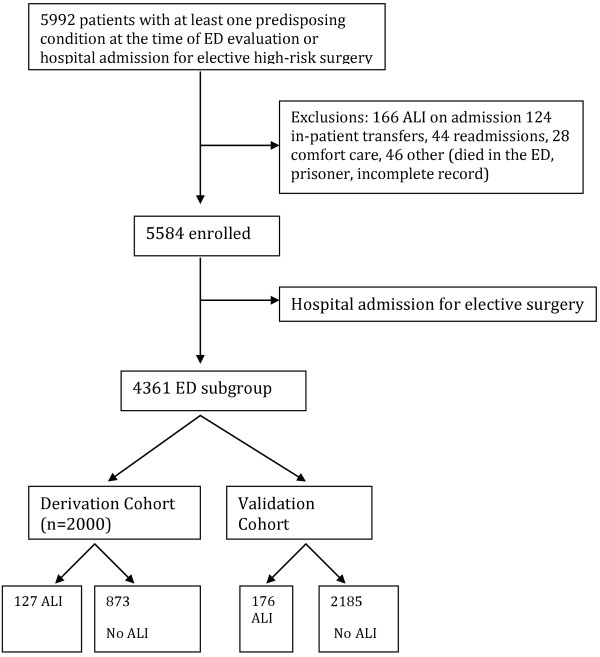
Study schematic.

Twenty-two centers screened 5,992 adult patients, of whom 4,361 were admitted from the emergency department and had at least one ALI risk factor. One hundred sixty-six patients were excluded with ALI on presentation, ED death, or other criteria. Predisposing conditions (aspiration, pneumonia, sepsis, shock, high-risk and emergency surgery, and high-risk trauma: lung contusion, multiple rib fractures, traumatic brain injury, smoke inhalation, and near-drowning) and clinical and physiological risk factors associated with ALI development were identified (Figure 1).

The overall incidence of ALI in the ED subgroup was 7.0% (303/4,361). The incidence of ALI in the derivation and validation groups was 6.4% (127/2,000) and 7.5% (176/2,361), respectively, *p* = 0.15. There were no statistically significant differences detected between the derivation and validation cohorts (Table 1).

**Table 1:** Comparison of derivation and validation cohorts

	**Total**	**Derivation**	**Validation**	
Variable*	**(*n*** **= 4,361)**	**(*n*** **= 2,000)**	**(*n*** **= 2,361)**	***p*-value**
**Demographics**				
Median age	56.0 (41.0, 71.0)	56.0 (42.0, 71.0)	56.0 (41.0, 71.0)	0.596
Male, no. (%)	2,422 (55.5%)	1,104 (55.2%)	1,318 (55.8%)	0.680
Caucasian, no. (%), *n* = 4,220	2,608 (61.8%)	1,193 (61.6%)	1,415 (62.0%)	0.765
Admission source, no. (%) *n* = 4,311				0.131
Home	3,331 (77.3%)	1,557 (78.8%)	1,774 (76.0%)	
Nursing facility	338 (7.8%)	151 (7.6%)	187 (8.0%)	
Outside ED	440 (10.2%)	183 (9.3%)	257 (11.0%)	
Other	202 (4.7%)	85 (4.3%)	117 (5.0%)	
APACHE II	10.0 (6.0, 15.0)	10.0 (6.0, 15.0)	10.0 (5.0, 15.0)	0.770
**Predisposing conditions**				
Shock	395 (9.1%)	180 (9.0%)	215 (9.1%)	0.903
Aspiration	210 (4.8%)	92 (4.6%)	118 (5.0%)	0.541
Sepsis	1,806 (41.4%)	856 (72.8%)	950 (70.2%)	0.087
Pancreatitis	323 (7.4%)	140 (7.0%)	183 (7.8%)	0.345
Pneumonia	1,227 (28.1%)	568 (28.4%)	659 (27.9%)	0.721
High-risk trauma				
Traumatic brain injury	490 (11.2%)	214 (10.7%)	276 (11.7%)	0.302
Smoke inhalation	27 (0.6%)	10 (0.5%)	17 (0.7%)	0.356
Near drowning	3 (0.1%)	2 (0.1%)	1 (0.0%)	0.597
Lung contusion	188 (4.3%)	87 (4.4%)	101 (4.3%)	0.907
Multiple fractures	330 (7.6%)	141 (7.1%)	189 (8.0%)	0.235
High-risk surgery				
Thoracic (noncardiac)	5 (0.1%)	3 (0.2%)	2 (0.2%)	0.526
Orthopedic spine	17 (0.4%)	6 (0.3%)	11 (0.5%)	0.381
Acute abdomen	295 (6.8%)	133 (6.7%)	162 (6.9%)	0.782
Cardiac surgery	20 (0.5%)	6 (0.3%)	14 (0.6%)	0.154
Aortic vascular	14 (0.3%)	5 (0.3%)	9 (0.4%)	0.445
**Risk modifiers**				
Alcohol abuse	421 (9.7%)	191 (9.6%)	230 (9.7%)	0.831
Obesity	1,020 (29.1%)	456 (28.5%)	564 (29.6%)	0.451
Chemotherapy	158 (3.6%)	82 (4.1%)	76 (3.2%)	0.121
Diabetes mellitus	1,042 (23.9%)	485 (24.3%)	557 (23.6%)	0.612
Smoking (*n* = 4019)				0.892
None	2,060 (51.3%)	932 (50.9%)	1,128 (51.6%)	
Former	888 (22.1%)	408 (22.3%)	480 (22.0%)	
Active	1,071 (26.7%)	493 (26.9%)	578 (26.4%)	
Emergency surgery	339 (7.7%)	154 (7.7%)	185 (7.8%)	0.868
RR	20.0 (18.0, 24.0)	20.0 (18.0, 24.0)	20.0 (18.0, 24.0)	0.658
Tachypnea	315 (7.6%)	145 (7.6%)	170 (7.6%)	0.948
SpO_2_	95.6 (95.4, 95.7)	95.2 (95.2, 95.7)	95.7 (95.5, 95.9)	0.068
SpO_2_ > 95%	2,662 (62.3%)	1,203 (61.4%)	1,459 (63.1%)	0.254
FiO_2_	0.2 (0.2, 0.3)	0.2 (0.2, 0.3)	0.2 (0.2, 0.3)	0.561
FiO_2_ > 0.35, no. (%)	841 (19.3%)	381 (19.1%)	452 (19.1%)	0.937
Albumin level	3.5 (2.9, 4.0)	3.5 (3.0, 4.0)	3.5 (2.9, 4.0)	0.068
Hypoalbuminemia	945 (47.1%)	414 (45.4%)	531 (48.5%)	0.167
pH median	7.4 (7.3, 7.4)	7.4 (7.3, 7.4)	7.4 (7.3, 7.4)	0.632
Acidosis (pH <7.35)	476 (45.9%)	206 (43.6%)	270 (47.8%)	0.173
**Outcome**				
ALI/ARDS	303 (7.0%)	127 (6.4%)	176 (7.5%)	0.153

### Derivation of prediction rule

The weighting of EDLIPS points was adjusted based on the multivariate logistic regression analysis in the derivation cohort of 2,000 randomly selected patients and was validated in the remaining (2,361) patients (Table 2).

**Table 2:** EDLIPS points derived and assigned by weight

**Predispositions**	**Estimate**	**95% CI**		**p-value**	**EDLIPS Points**
Male gender	0.512	0.089	0.935	0.018	1
Aspiration	0.900	0.204	1.595	0.011	2
Pneumonia	0.550	0.052	1.047	0.030	1
Sepsis	0.500	0.031	0.969	0.037	1
Shock	0.959	0.406	1.512	0.001	2
Lung contusion	0.823	−0.032	1.679	0.059	1
Smoke inhalation	1.505	−0.100	3.109	0.066	1.5
Long bone fractures	1.122	0.341	1.904	0.005	2
Brain injury	1.103	0.426	1.780	0.001	2
Cardiac surgery	2.584	0.619	4.549	0.010	5
Aortic surgery	2.619	0.190	5.049	0.035	5
Spine surgery	2.727	0.623	4.832	0.011	5
Acute abdomen	1.272	0.506	2.038	0.001	2.5
**Risk modifiers**					
Diabetes mellitus	−0.381	−0.896	0.133	0.146	−0.5
Cirrhosis	0.928	−0.078	1.934	0.071	1
Chemotherapy	1.181	0.405	1.957	0.003	2
Obesity (BMI >30)	0.795	0.352	1.237	0.000	1.5
Acidosis (pH <7.35)	0.852	0.348	1.357	0.001	2
FiO_2_ > 0.35 (>4 l/min)	0.904	0.443	1.365	0.000	2
Albumin <3.5	0.792	0.359	1.226	0.000	1.5
SpO_2_ < 95%	0.733	0.317	1.148	0.001	1.5
Excluded variables*					
Pancreatitis	0.273	−0.866	1.413	0.638	-
Thoracic surgery	1.187	−2.476	4.849	0.525	-
Near drowning^a^	14.509	−7692.1	7721.1	0.997	-
Alcohol abuse^a^	0.099	−0.618	0.817	0.786	-
Smoking	−0.054	−0.504	0.397	0.816	-
Tachypnea	0.074	−0.596	0.745	0.828	

*Variables were removed secondary to minimal effect size; ^a^fewer than ten patients with variable.

Emergency department admissions requiring cardiac, aortic, or spine surgeries had the highest assignments of EDLIPS points, conferring the highest associated risk for ALI development. Other factors were also observed to have significant effect including the presentation of acute abdomen, multiple long bone fractures, traumatic brain injury, aspiration, shock, chemotherapy, acidosis, or an oxygen requirement of >0.35 FiO_2_. A modest influence on progression to ALI was observed with male gender, pneumonia, sepsis, lung contusion, obesity, hypoalbuminemia, and hypoxemia. In contrast, the pre-admission diagnosis of diabetes mellitus conferred protection from ALI with an assignment of negative 0.5 points. The EDLIPS model calculation worksheet and examples of how to calculate the score are presented. (Table 3).

**Table 3:** EDLIPS score calculation worksheet

**Using Table 2 examples**	
i.	Patient with history of cirrhosis with septic shock from pneumonia requiring FIO_2_ > 0.35 in the emergency room:
	Sepsis + shock + pneumonia + cirrhosis + FIO_2_ > 0.35
	1 + 2 + 1 + 1 + 2 = 7
ii.	Motor vehicle accident with traumatic brain injury, lung contusion, and shock requiring FiO_2_ > 0.35
	Brain injury + lung contusion + shock + FiO_2_ > 0.35
	2 + 1 + 2 + 2 = 7
iii.	Patient with history of diabetes mellitus presents with urosepsis, acidosis and shock
	Sepsis + shock + acidosis + diabetes
	1 + 2 + 2–0.5 = 4.5

In this cohort the lowest and highest EDLIPS scores achieved were −0.5, 15.

**Figure 2:**
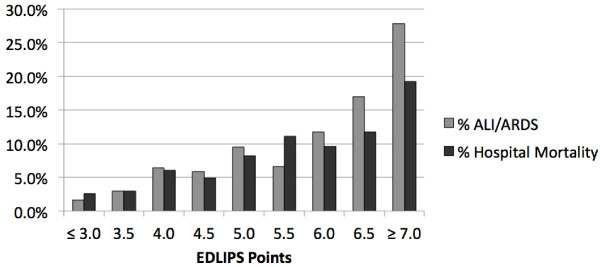
**Frequency of ALI/ARDS development and hospital mortality according to EDLIPS value (*n*** **= 4,361).**

The model was well calibrated in both training and testing data sets. EDLIPS scores ranged from −0.5 to 15, median 3.5 (IQR: 2.0, 5.0). Among patients who ultimately developed ALI, the median LIPS score was 6.5 (IQR: 4.5, 8.0) compared to those who did not, median 3.5 (IQR: 2.0, 5.0). Overall, the incidence of ALI increased with increasing LIPS score. EDLIPS score ≥7 was associated with a 27.9% frequency of ALI development, while a score of ≤3 had a frequency of 1.7%. Hospital mortality was 19.2% for those with an LIPS score ≥7 compared to 2.6% for those with a score of ≤3 (Figure 2).

**Figure 3:**
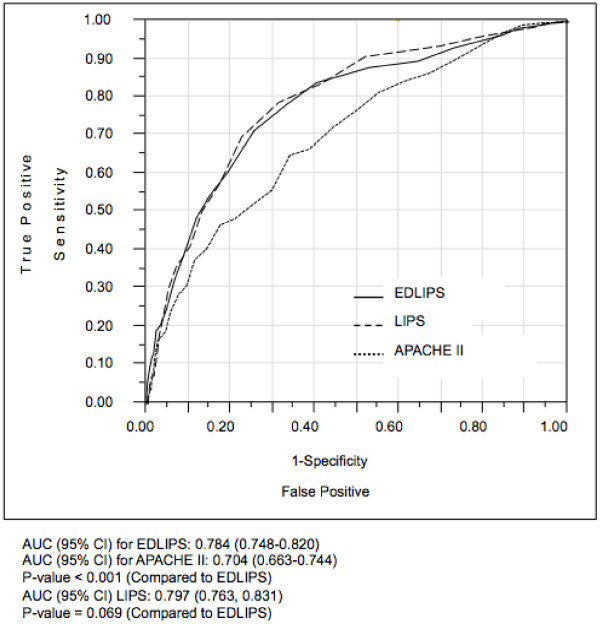
**Receiver operating characteristic curves for EDLIPS points, LIPS points, and APACHE II in the validation group (*n*** **= 2361).** AUC (95% CI) for LIPS: 0.784 (0.748-0.820), AUC (95% CI) for APACHE II: 0.704 (0.663-0.744), P-value < 0.001 (Compared to LIPS), AUC (95% CI) Original LIPS: 0.797 (0.763, 0.831), P-value = 0.069 (Compared to LIPS).

EDLIPS discriminated patients who developed ALI from those who did not with an AUC of 0.78 (95% CI 0.75, 0.82) (Figure 3). At an EDLIPS cutoff of 5.0 (range −0.5-15), positive and negative likelihood ratios (95% CI) for ALI development were 2.74 (2.43, 3.07) and 0.39 (0.30, 0.49), respectively, with a sensitivity of 0.72 (0.64, 0.78), specificity of 0.74 (0.72, 0.746), positive predictive value of 0.18 (0.15, 0.21), and negative predictive value of 0.97 (0.96, 0.98) (Table 4).

**Table 4:** **Sensitivity analysis: EDLIPS performance at different cutoff points (*n*** **= 2,361)**

	**EDLIPS cutoff points**		
**EDLIPS performance**	≥5*	**≥4**	**≥6**
Prevalence of ALI/ARDS (95% CI)	0.075 (0.065, 0.086)	0.075 (0.065, 0.086)	0.075 (0.065, 0.086)
Sensitivity (95% CI)	0.716 (0.643, 0.781)	0.852 (0.791, 0.901)	0.557 (0.480, 0.632)
Specificity (95% CI)	0.738 (0.719, 0.757)	0.592 (0.571, 0.613)	0.848 (0.832, 0.862)
Positive predictive value	0.181 (0.153, 0.211)	0.144 (0.123, 0.167)	0.227 (0.189, 0.270)
Negative predictive value	0.970 (0.961, 0.978)	0.980 (0.971, 0.987)	0.960 (0.950, 0.968)
Likelihood ratio (+)(95% CI)	2.735 (2.434, 3.073)	2.088 (1.928, 2.261)	3.654 (3.099, 4.308)
Likelihood ratio (−)(95% CI)	0.385 (0.304, 0.487)	0.250 (0.175, 0.357)	0.523 (0.443, 0.618)
C-statistic (95% CI)	0.727 (0.692, 0.762)	0.722 (0.694, 0.750)	0.702 (0.748, 0.820)

*Depicts optimal cutoff.

In contrast, the APACHE II score had limited prognostic accuracy for ALI development of AUC 0.70 (95%CI 0.66, 0.74),*p* value <0.001, compared to EDLIPS (Figure 3).

In-hospital mortality was higher for patients with ALI compared to those without (27.7% vs 4.6%, *p* < 0.001). The unadjusted odds ratio of death from ALI/ARDS is 7.90 (95% CI:5.90,10.56),*p* < 0.001. After adjusting for both EDLIPS and APACHE II scores, the odds ratio for hospital mortality is 1.29 (95% CI: 1.23, 1.36), *p* < 0.001, and 1.17 (95%CI: 1.15, 1.19), *p* < 0.001, respectively.

When compared to the performance of the EDLIPS score, there was no statistically significant difference from the original LIPS in predicting the cohort of patients who developed ALI: original LIPS AUC [0.80 (95% CI: 0.76, 0.83) *p* = 0.07].

## Discussion

Since the 1990s, US hospitals have experienced a 55% increase in critically ill presentations to EDs. With over 110 million visits a year, the trend in emergency medicine investigations has been directed at early risk stratification and goal-directed care, particularly in the critically ill. Hou et al. report that in the at-risk population of ED patients, up to 7% develop ALI within a median of 2 days (IQR 2–5) [22].

ALI can represent a devastating pulmonary process associated with increased length of stay, costs, and long-term poor outcomes [23,24]. Moreover, it represents a disease that has the potential to impart a burden across a younger and healthier population than previously recognized [25]. The median age of this EDLIPS cohort was 56 years. In one 5-year longitudinal trial, survivors complained of persistent neuropsychological impairment and high personal medical expenditures [26]. Persistent exercise limitation and pulmonary fibrosis are common [26-28]. Preventing ALI and progression to ARDS has the potential to facilitate the return of viable at-risk patients back to their communities with the capacity to provide meaningful contributions to society.

This preliminary study suggests that the risk of progression to ALI may be ascertained using the EDLIPS. The score and consequent degree of risk varies according to the type and number of predisposing conditions. The finding that emergency high-risk surgeries, traumatic injuries, and shock were strong indicators is consistent with the literature, which cites a high incidence of ARDS in these populations [29]. Our model also found that the requirement of >4 l/min and chemotherapy was a determinate in progression to ALI, similar to previously published work by Levitt et al. [30].

Other predisposing conditions analyzed, such as near drowning, may have also proven to be strong predictors; however, there were too few cases to reliably detect an effect. It remains unclear, however, why the existence of diabetes mellitus confers protection to patients. Previous investigations have noted a lower incidence of ALI among diabetics compared to their non-diabetic cohorts. Honiden and Gong suggest that hyperglycemia as well as the therapeutic interaction of medications may alter the inflammatory response associated with ALI/ARDS development [21].

It is interesting that conditions such as pneumonia and pancreatitis were not stronger predictors in this cohort. The study was not designed to assess the severity of illness. The high number of patients presenting with less complicated disease may have dampened any resulting signal from severe cases of pneumonia or acute pancreatitis. However, the concomitant existence of hypoxemia, high oxygen requirement, and/or acidosis, for example, would increase a patient’s risk for developing ALI.

When compared to the previously published scoring methodology of LIPS, the EDLIPS affords a number of advantages. It identifies patients who are at risk for ALI from a broader scale of potential presenting symptoms and predisposing conditions in the ED. While EDLIPS did not out-perform original LIPS, its ability to discriminate patients who would go on to develop ALI is comparable in this study. Moreover, EDLIPS is derived from a targeted population of ED patients and lacks the heterogeneity included in the original LIPS cohort of patients admitted from the ED and patients undergoing high-risk elective surgeries.

This affords the potential for EDLIPS to discern factors unique to the ED population. It is notable that in one 8-year longitudinal study of ARDS, the hospital and ICU populations experienced a dramatic reduction in ARDS attributed to clinical interventions, while the incidence of early onset ARDS within 6 h of ED admission remained unchanged [31]. This suggests potential differences in the mechanistic pathways in the development of ALI.

This EDLIPS scoring method is designed for the ED setting utilizing routinely available clinical variables that can readily be identified upon presentation for risk stratification predicting progression to ALI and in-patient mortality. Moreover, the scoring system has the potential to allow for the investigation of preventive measures in the emergency department. While the authors acknowledge APACHE II was not designed or intended to predict ALI, it is a broadly recognized assessment tool utilized among critically ill patients. APACHE II is consistently referenced as the model when validating the performance of customized scores in a heterogeneous population of critically ill patients. As such, it is not surprising that it lacks discriminating capacity in predicting ALI when compared to EDLIPS. However, it is notable that EDLIPS with increasing scores predicted an increasing trend of mortality, suggesting an increased severity of illness, for which purpose the APACHE had been originally designed. Moreover, from the standpoint of clinical practice, when compared to EDLIPS, APACHE II requires 12 separate physiological data points and a sophisticated calculation scheme to derive a score over a 24-h period. Its use in the ED is not feasible [32,33].

A frequent consequence of critical illness in the emergency department is intubation and mechanical ventilation, a hallmark of ED resuscitation and a requisite component of the clinical management of the ALI/ARDS patient. Studies suggest that early ventilator settings influence downstream outcome of critically ill patients [34]. The application of mechanical ventilation can induce pulmonary damage by means of a process termed ventilator-associated lung injury (VALI). Both animal and human studies demonstrate upregulation of inflammatory cytokines, which compromise the alveolar capillary membrane when increased volumes are applied to the lung parenchyma. This mechanical stress can produce a stimulus that induces the transformation of a normal lung to a lung with histological appearance indistinguishable from ALI induced by sepsis, shock, or pneumonia [35-38]. The clinical impact of high tidal volume ventilation was underscored by the Acute Respiratory Distress Syndrome Network study. Utilizing the lower tidal volume, mortality from ARDS was reduced from 39.8% to 31% [15]. To date, the primary strategy proven to be effective at reducing mortality from ALI is low tidal volume ventilation by targeting a reduction in VALI. Even more compelling is an investigation by Determann et al. demonstrating that randomly selected patients without ALI placed on mechanical ventilation with low tidal volumes of 6 ml/kg were less likely to develop ALI than those placed on 10 ml/kg predicted body weight (2.6%, 13.5%, *p* = 0.01 [39]).

Assuming preventive strategies are identified, the EDLIPS has the potential to result in substantial morbidity and mortality reduction as well as cost savings. Specifically, an EDLIPS of ≥5 should prompt the clinician team to closely monitor the patient and communicate the potential need to address acute changes in respiratory status to the receiving service; this in turn would allow for the institution of preventive measures.

The transition of ALI studies from the ICU to the ED population may not only be prudent but obligatory as studies demonstrate the preponderance of ARDS cases are likely to stem from ED admissions secondary to insults exposed in the community. Hence, investigations evaluating the use of antiplatelet and statin therapies, a low tidal volume ventilation strategy, and restrictive transfusion in the ED may indeed be needed to mitigate ALI development [40-43]. Designed as a bundle, these interventions have the potential to curb the progression of illness in a patient identified at risk when instituted early.

### Limitations

While the EDLIPS model did accurately identify most ALI patients at higher LIPS scores, it is notable that the model has a modest AUC. So, while a robust negative predictive value of 0.97 renders the model useful in screening patients with low risk for ALI, the weaker positive predictive value does lack precision in identifying those at high risk for ALI. Alternatively, the use of a higher threshold score may enhance the model’s performance in the clinical setting.

### Future directions

This study represents an initial attempt to refine a scoring methodology of emergency department patients for the purpose of predicting ALI development. External validation will be necessary to determine whether EDLIPS can be generalized to clinical practice. Moreover, it is unclear what specific impact the implementation of this scoring system will have on physician practice, patient outcomes, or resource utilization. Further studies will be needed to assess the application of this scoring system in conjunction with outlined strategies known to have an impact on clinical parameters in patients at risk for ALI.

## Conclusions

In this study, we describe a variation on a novel scoring method that screens and stratifies patients at greatest risk for developing ALI in the ED. Although the overall performance is modest, an excellent negative predictive value makes it a useful screening tool. EDLIPS performance was similar to the original LIPS model and significantly better than APACHE II in predicting ALI development. Confirmation of these results in other ED populations and the identification of additional risk factors could aid both the identification of susceptible individuals and the targeting of therapies.

## Abbreviations

ALI: Acute lung injury; ARDS: Acute Respiratory Distress Syndrome; APACHE: Acute Physiology and Chronic Health Evaluation; ED: Emergency department; EDLIPS: Emergency Department Lung Injury Prediction Score; ICU: Intensive care units; LIPS: Lung Injury Prediction Score; AUC: Receiver-operating characteristic curve; PEEP: Positive end expiratory pressure; VALI: Ventilator-associated lung injury.

## Competing interests

Dr. Frendl provided internal funding for research staff and biostatistic support from STAR Center, Brigham and Women’s Hospital, Boston, MA. Dr. Gajic is supported in part by grants from the National Heart, Lung, and Blood Institute HL78743-01A1; National Center for Research Resources 1 KL2 RR024151. Dr. Gentile is supported in part by a grant from the National Institute of Neurological Disorders and Stroke 5U10NS059039. The rest of the authors have no disclosures or conflict of interest.

## Authors’ contributions

ME, PCH, OG, and NTG conceived the study and designed the trial. PCH and GF obtained research funding and resources. ME, PCH, OG, and NTG supervised the conduct of the trial and data collection. AM and OG managed the data, including quality control. AM, ME, PCH, and OG provided statistical advice on study design and analyzed the data. ME drafted the manuscript, and all authors contributed substantially to its revision. ME takes responsibility for the paper as a whole.
